# Interactions of Ionic Liquids and Spirocyclic Compounds with Liposome Model Membranes. A Steady-State Fluorescence Anisotropy Study

**DOI:** 10.1038/s41598-019-53893-w

**Published:** 2019-12-04

**Authors:** Antti H. Rantamäki, Wen Chen, Paulus Hyväri, Jussi Helminen, Gabriel Partl, Alistair W. T. King, Susanne K. Wiedmer

**Affiliations:** Department of Chemistry, A.I. Virtasen aukio 1, University of Helsinki, P.O. Box 55, FI-00014 Helsinki, Finland

**Keywords:** Chemical biology, Chemical safety, Green chemistry, Biophysical chemistry, Lipids, Biological fluorescence, Membrane biophysics, Membrane structure and assembly, Permeation and transport, Biophysical chemistry, Membranes

## Abstract

Understanding the toxicity of ionic liquids (ILs) is crucial in the search of greener chemicals. By comparing *in vivo* toxicity and *in vitro* interactions determined between compounds and biomimetic lipid membranes, more detailed toxicity vs. structure relation can be obtained. However, determining the interactions between non-surface-active compounds and liposomes has been a challenging task. Organisational changes induced by ILs and IL-like spirocyclic compounds within 1,6-diphenyl-1,3,5-hexatriene-doped biomimetic liposomes was studied by steady-state fluorescence anisotropy technique. The extent of organisational changes detected within the liposome bilayers were compared to the toxicity of the compounds determined using *Vibrio Fischeri* bacteria. Four liposome compositions made of pure 1-palmitoyl-2-oleyl-*sn*-glycero-3-phosphocoline (POPC) and mixtures of POPC, 1-palmitoyl-2-oleyl-*sn*-glycero-3-phosphoserine (POPS), and cholesterol (Chol) were tested as biomimetic models. Changes observed within the POPC/POPS/Chol 55:20:25 bilayers correlated the best with the toxicity results: ten out of twelve compounds followed the trend of *increasing bilayer disorder – increasing toxicity*. The study suggests that the toxicity of non-surface-active compounds is dependent on their ability to diffuse into the bilayers. The extent of bilayer’s organisational changes correlates rather well with the toxicity of the compounds. Highly sensitive technique, such as fluorescence anisotropy measurements, is needed for detecting subtle changes within the bilayer structures.

## Introduction

Ionic liquids (ILs) and other IL-like organic salts, such as spirocyclic compounds can be utilised in numerous applications, *e.g*. as solvents for wood and other biomass processing applications^1^. In the search of greener solvents or processes, the toxicity and biological activity of these chemicals plays a crucial role^2^. According to previous studies, it is evident that long alkyl chains attached either to the anion or cation of the ILs, are directly correlated to high toxicities of the compounds^3–14^. The higher toxicities caused by the long alkyl chains can be explained by the increased surface activity and lipophilicity of the ion and its resulting ability to efficiently permeate into the membrane bilayers^6,8,9,13,14^. In general, the (relative) toxicities of the compounds are therefore easy to predict, based on the alkyl chain length – the longer the chain, the higher the toxicity.

Phospholipid bilayers have been used as well-accepted models of natural biomembranes^15^. In the aforementioned studies^6,8,9^, permeation of the surface-active compounds into such biomimetic liposomes was detected utilizing differential scanning calorimetry (DSC). However, certain low-toxicity compounds, such as 1,5-diazabicyclo[4.3.0]non-5-enium acetate ([DBNH][OAc]), did not induce any detectable changes within the bilayers when studied with DSC, even at 400 mM concentration^9^. Similarly, no effect was detected when the interaction of [DBNH][OAc] with biomembranes was studied using nanoplasmonic sensing (NPS) at 250 mM concentration^16^. However, this does not necessarily mean that such compounds would not interact with, or permeate into the bilayer but rather suggests that the techniques used in the previous studies may not be suitable for such determinations. DSC is may not sensitive enough to detect such changes and increasing the concentration of the compounds is not practical. With NPS the dynamic character of the technique might slow down the diffusion and permeation into the membranes. However, it is expected that as relatively small molecules, they could permeate through the plasma membranes into the cell without active transport – despite the fact that this could not be detected in the previous studies. According to molecular simulations investigating permeation of relatively small and hydrophilic cholinium glycinate into model lipids layers, the small cations permeated into the polar head group region of the lipid bilayer, whereas the anion showed only very superficial interaction with the model membrane^17^. However, when more lipophilic ILs were investigated using similar models, the compounds were permeating deeper into the membrane^13,14^.

In the present study, we investigated the potentially toxic interactions of nine organic salts (Table 1) with model lipid bilayers. The compounds consisted of four heterobicyclic and two non-cyclic mesylate ILs and of three spirocyclic acetates. These compounds of interest are non-surface-active (other than the reference compound, *vide infra*) and are expected to have low toxicities, based on their structures and our experiences from previous work^9^. Similar compounds to the ones investigated here have not shown any relevant correlation with the toxicity data^9^. The only common denominator for compounds of low toxicity is that no interaction with the biomimetic membranes have been detected. Since the toxicity is dosage-dependent, even the compounds classified as “harmless or practically harmless” (EC_50_ > 100 mg/L, as described in a previous study^7^) become toxic when the concentration is high enough. Therefore, these low-toxicity compounds need higher concentration to permeate into the membranes or to access into the organism in order to exhibit any toxic effect. The purpose of the present study was to investigate if such interactions could be detected using a more sensitive technique.

**Table 1:** Compounds, their abbreviations and molecular structures.

Spirocyclic compounds	Abbreviation	Cation structure
5-Azoniaspiro[4.5]decane acetate	[4.5][OAc]	
6-Azoniaspiro[5.5]undecane acetate	[5.5][OAc]	
6-Azoniaspiro[5.6]dodecane acetate	[5.6][OAc]	
**Ionic liquids**	**Abbreviation**	**Cation structure**
1,5-Diazabicyclo[4.3.0]non-5-enium mesylate	[DBNH][OMs]	
1,8-Diazabicyclo[5.4.0]undec-7-enium mesylate	[DBUH][OMs]	
5-Methyl-1,5,7-triazabicyclo[4.3.0]non-6-enium & 7-Methyl-1,5,7-triazabicyclo[4.3.0]non-5-enium mesylate	[MTBNH][OMs]	
7-Methyl-1,5,7-triazabicyclo[4.4.0]dec-5-enium mesylate	[MTBDH][OMs]	
Triethylammonium mesylate	[TEAH][OMs]	
1,1,3,3-Tetramethylguanidinium mesylate	[TMGH][OMs]	
**Reference compounds**	**Abbreviation**	**Structure**
Methyltrioctylphosphonium acetate (reference compound)	[P_8881_][OAc]	
Sodium acetate (reference compound)	NaOAc	
Sodium mesylate (reference compound)	NaOMs	

The technique used in the present study was steady-state fluorescence anisotropy spectroscopy (*fluorescence anisotropy* or *anisotropy* in the following text). The technique was utilised to detect organisational changes within the lipid bilayer using a fluorescent 1,6-diphenyl-1,3,5-hexatriene (DPH) probe. DPH is advantageous in the present application due to its high quantum yield and its ability to indicate subtle changes in the lipid acyl chain organisation. For general reviews on the technique, see Shinitzky & Barenholz^18^ and Lentz^19^. The rod-like hydrophobic probe is rotating within the bilayer structure by Brownian motion and it is located between the hydrophobic acyl chains of the lipids. When the probe-containing liposomes are illuminated with UV-light at a fixed wavelength and a fixed level of polarized light, the probe is first excited and subsequently emits light that is partly depolarized, due to the molecular rotations of the probe. This depolarization can be measured as a numerical value of anisotropy (*r)*, as shown by Eq. 1.

1 $$r=\frac{{I}_{||}-{I}_{\perp }}{{I}_{T}}$$

where I_||_ and I_⊥_ are the intensities of emitted light parallel and perpendicular to the plane of excitation, respectively. I_T_ is the total intensity of emitted light.

Based on the local environment defined by the lipid chain order, the probe’s molecular rotation is restricted: the more organised the bilayer, the less rotational freedom. Based on the recorded anisotropy values, for instance, for pure liposome and toxicant-exposed liposomes, the toxicant-induced changes in the lipid layer organisation can be assessed. Organisational changes within the lipid acyl chain region further affect the entire viscosity (*i.e*. fluidity) of the membrane. Fluidity of the membrane further affects the homeostasis of the living organisms and therefore may be linked to the toxicity of compounds. Also, if the compounds are able to permeate into the bilayer, they may also be able to diffuse through the bilayer into the cell, and, thus, may also induce their toxic effect within the cell. Due to the low DPH:lipid ratio (1:499 in the present study) the rod-like probes only interfere locally with the bilayer organisation and thus, the probes’ interference does not have any practical impact on the bilayer organisation, particularly in fluid membranes such as those used in the present study^20,21^.

In addition to the high sensitivity, another benefit of the technique is that it also allows for modifications of the liposome lipid composition. This is not possible with DSC, which is dependent on the main phase-transition behaviour of the bilayer, and therefore the selection of lipids for this purpose is more limited^8,9^. In addition, the phase of the lipid layer is known to affect the permeability of the lipid layer for small molecules^22^. Particularly around the main phase transition temperature, the membranes turn leaky for small molecules^23^. The fluorescence anisotropy technique therefore facilitates the use of fluid lipid bilayers, which is highly preferred as natural membranes are mostly in a fluid phase and condensed phases appear only locally.

Interactions between ILs and liposomes have been investigated before by using fluorescence anisotropy measurements^24^. However, to our best knowledge there is no previous attempts to show correlation between compounds’ toxicity and their liposome interactions using this technique.

## Results and Discussion

**Figure 1:**
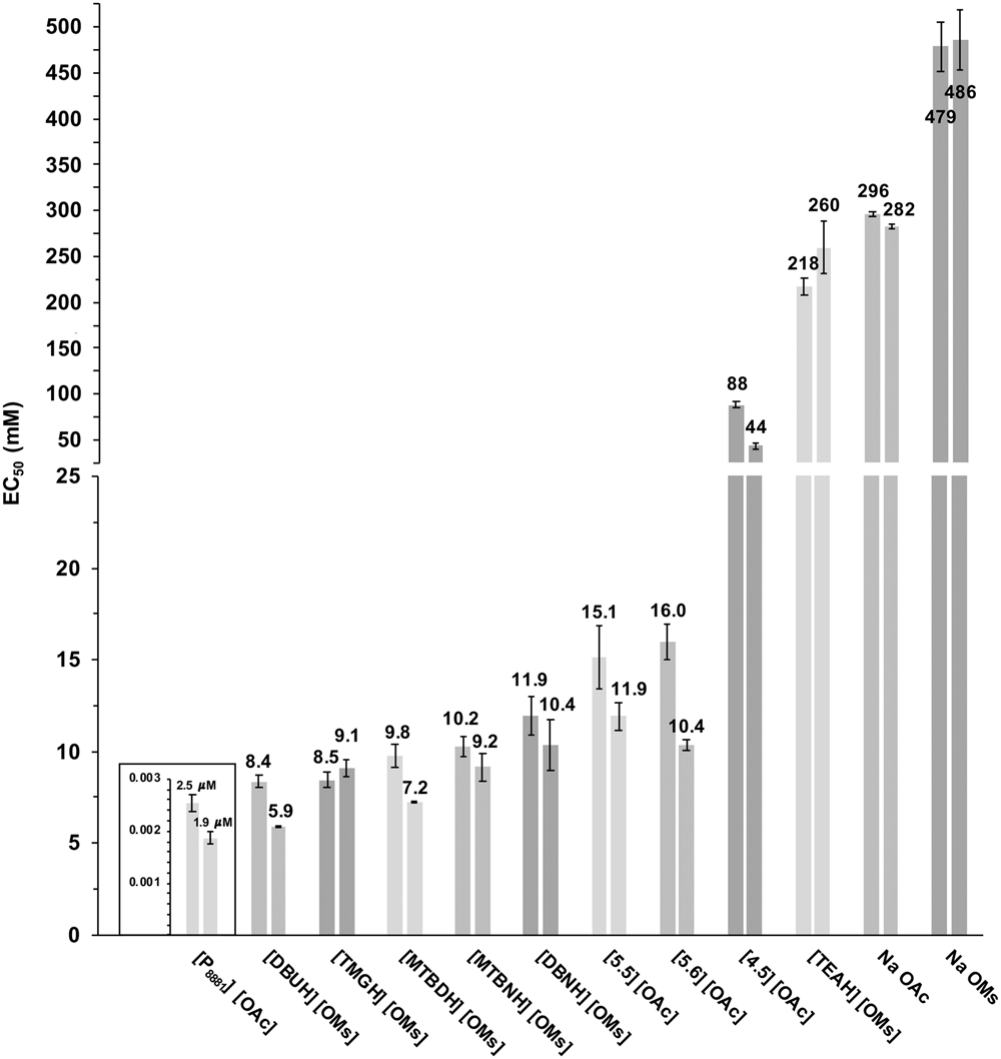
The median effective concentrations EC_50_ for the ionic liquids and the spirocyclic compounds was measured using *Vibrio Fischeri* bacteria. The EC_50_ values are the mean of two duplicate measurements ± standard deviation. Note the differing scale for [P_8881_][OAc] and the change of the scale along the vertical axis. Left-hand bar, 5 min exposure; Right-hand bar, 15 min exposure.

In this study, the toxicity of certain organic salts purposed for biomass processing was investigated (see Table 1 for structures). The main goal of the study was to investigate if there is a correlation between the compounds’ EC_50_ values measured using *Vibrio Fischeri* bacteria (Fig. 1) and their potential interactions with biomimetic liposomes measured using fluorescence anisotropy.

The bacteria were incubated for 5 and 15 minutes with the compounds to investigate the time-dependency (and to provide both values for future reference) of the toxic effect. As expected, the 15 min values are mostly smaller than the 5 min values, triethylammonium mesylate [TEAH][OMs] being the one exception. However, the difference between the 5- and 15-min values is miniscule when the error bars are considered. Overall, the order of toxicity between the compounds does not dramatically change with incubation time. The EC_50_ value (2–3 µM) for the positive control methyltrioctylphosphonium acetate ([P_8881_][OAc]) was obtained from Russo *et al*.^25^. In the following discussion, the 5 min EC_50_ values and the anisotropy values are compared, since also in anisotropy measurements the incubation time of the liposomes and compounds of interest was 5 min. The EC_50_ values, in mass concentration, are shown in Fig. S1 (supplementary Information).

**Figure 2:**
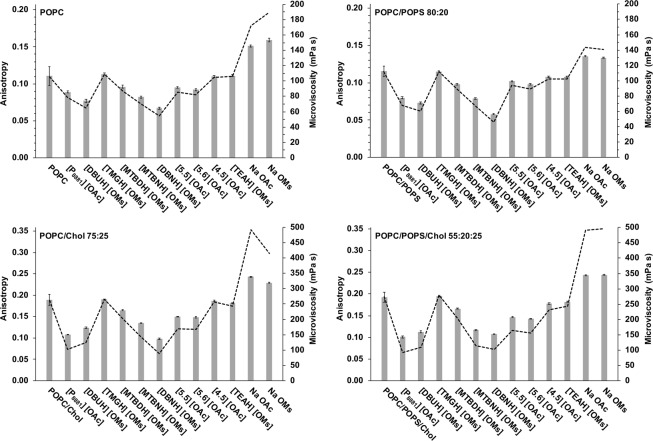
Anisotropy (bars) and microviscosity (dotted line) values for each of the four liposome compositions before (leftmost bar) and after interaction with the compounds. The anisotropy values are the mean of ten measurements ± standard deviation. The anisotropy shown for each pure liposome is the mean of all daily control values measured during the study. This results in larger standard deviations relative to the others. The error bars from microviscosity values were omitted for clarity. Note the differing scales of the upper and lower panels.

A reference anisotropy value for each pure liposome was determined daily. This was performed in order to normalise the liposome batch-to-batch and liposome-aging-dependent variation of the anisotropy values. The anisotropy was measured ten times and the mean ± standard deviation was calculated. Subsequently, the pure liposomes and the compounds under investigation were mixed and incubated at room temperature for 5 min, before measuring the anisotropy. Similar to the reference measurements, the fluorescence anisotropy value after incubation with the ILs was recorded 10 times and the mean ± standard deviation was calculated. The anisotropy values are presented in Fig. 2.

Three lipids, namely 1-Palmitoyl-2-oleyl-*sn*-glycero-3-phosphocoline (POPC), 1-palmitoyl-2-oleyl-*sn*-glycero-3-phosphoserine sodium salt (POPS) and bovine wool cholesterol (Chol), and four lipid compositions were utilised in preparation of the liposomes, namely POPC, POPC/POPS 80:20, POPC/Chol 75:25 and POPC/POPS/Chol 55:20:25 (Table 2). POPC was selected as the main neutral lipid covering a minimum of 55 mol% of lipids in each liposome. POPS in 20 mol% was present in two compositions, in order to investigate the effect of anionic lipids in the liposome bilayer. Cholesterol at 25 mol% was present in two of the compositions, to better mimic natural plasma membranes and to investigate the impact of cholesterol on the interactions.

**Table 2:** Liposome compositions. The molar ratio of DPH:lipid in each liposome was 1:499.

Liposome	POPC (mol%)	POPS (mol%)	Cholesterol (mol%)
POPC	100	—	—
POPC/POPS	80	20	—
POPC/Chol	75	—	25
POPC/POPS/Chol	55	20	25

POPC, 1-palmitoyl-2-oleyl-*sn*-glycero-3-phosphocoline; POPS 1-palmitoyl-2-oleyl-*sn*-glycero-3-phosphoserine, Chol; Cholesterol.

Compound concentration of 50 mM was selected by trial and error, since such concentration seemed to induce detectable changes in anisotropy values for all compounds. This enabled direct comparison of the interaction between the compounds. In addition to the nine compounds of interest, three reference compounds were utilised: [P_8881_][OAc] as a highly toxic ionic liquid (EC_50_ = 2–3 µM)^25^ and NaOAc and NaOMs to better investigate the effect induced by the pure anions of the salts. The assumption here was that the sodium cation has minimal effect.

The aforementioned daily reference values for the pure liposomes resulted in mean anisotropy values of 0.111 ± 0.013 and 0.114 ± 0.007 for POPC and POPC/POPS, respectively (Fig. 2). A minimal difference between the values was expected, since the organisation of POPC and POPS into membranes is very similar, particularly regarding the acyl chain region. For the two cholesterol-containing liposomes, i.e. POPC/Chol and POPC/POPS/Chol, the anisotropy values were 0.188 ± 0.013 and 0.194 ± 0.013, respectively. Larger values, than those obtained for non-cholesterol containing liposomes, were expected, since the cholesterol molecules are located between lipid acyl chains and decrease the rotational freedom of the DPH probe. A general comparison of the anisotropy value profiles between the different liposome compositions shows that the profiles are rather similar (Fig. 2). After incubation with the compounds of interest, the POPC and POPC/POPS compositions showed very similar values of anisotropy and similarly the cholesterol-containing POPC/Chol and POPC/POPS/Chol compositions resembled each other. This is expected, since from lipid-acyl-chain point of view, the two non-cholesterol-containing liposomes are very similar. The same applies to the cholesterol containing compositions. The presence of negative charge in POPS does not considerably affect the order of the acyl chains, but may affect the permeation of the compounds as discussed later.

Equation 2 shows one form of the Perrin equation, which can be utilised to determine the apparent microviscosity *η* of a system by using fluorescence anisotropy measurements^18^.

2 $${(\frac{{r}_{0}}{r}-1)}^{-1}=\frac{\eta }{C(r)\cdot T\cdot \tau }$$

In this equation *r*_0_ is the fundamental anisotropy in the absence of rotational diffusion of the probe, i.e. the maximum anisotropy value achievable using the specific excitation wavelength. At 360 nm r_0_ = 0.362 for DPH^26^. *C(r)* relates the molecular shape and the location of the transition dipoles of the rotating probe into one parameter^18^. For spherical probes *C(r)* = *k/v*, where *k* is the Boltzmann constant and *v* is the effective volume of the probe. Therefore, *C(r)* would be constant for spherical molecules, but for other shapes, such as the rod-like DPH, the value is dependent on the experimental value of *r*. In contrast, as experimentally determined by Shinitzky and Barenholtz, the factor *C(r)· T·* τ = 240 mPa · s is relatively constant at 0–40 °C in membranes doped with DPH probe. T and τ are the temperature during the experiment and lifetime of the excited state, respectively. By substituting r_0_ = 0.362 and *C(r)· T·* τ = 240 mPa · s and by further rearranging of the Eqs. 2 and 3 is obtained.

3 $$\eta =\frac{240\,mPa\,s\cdot r}{0.362-r}$$

The equation gives the approximate apparent microviscosity (±15%) based on the recorded anisotropy values. The benefit of determining this is the hyperbolic growth of the apparent microviscosity relatively to anisotropy. This better shows the differences induced by the compounds in the bilayer organisation. The term *apparent microviscosity* is used since the freedom of rotation within the bilayer is spatially more restricted than, for instance, in other hydrophobic environments, such as in an oil. However, the term *microviscosity* is used in the following discussion when referring to the apparent microviscosity.

The microviscosity values are presented in Fig. 2 relative to the recorded anisotropy values. The microviscosity values for non-cholesterol-containing and cholesterol-containing liposomes were ~100 mPa·s and ~250 mPa·s, respectively (Fig. 2). After incubation with the compounds, the microviscosity values ranged from approximately 50 mPa·s to 500 mPa·s. Typically, the microviscosity of biological and artificial phospholipid membranes is 100–1000 mPa·s at 0–40 °C^18^. Values below 100 mPa·s are typical for highly unsaturated, *i.e*., highly disorganised lipid systems. In the present study, values < 100 mPa s are most likely caused by the permeation of large quantities of the compounds of interest into the bilayer. These compounds interfere with the network of interactions between the acyl chains and, consequently, the order of the bilayer decreases.

In order to compare the anisotropy values with the toxicity values in greater detail, the relative change in anisotropy *Δr* (%) was calculated as given by Eq. 4

4 $$\Delta r=\frac{{r}_{liposome+toxicant}-{r}_{pureliposome}}{{r}_{pureliposome}}$$

where *r*_*pure liposome*_ is the daily anisotropy value measured for each pure liposome and *r*_*liposome*+*toxicant*_ the anisotropy value measured after mixing the pure liposome with the compound of interest. In addition, the relative change in the microviscosity can be calculated but the outcome of the comparison would have been the same, so a more straightforward approach was selected.

**Figure 3:**
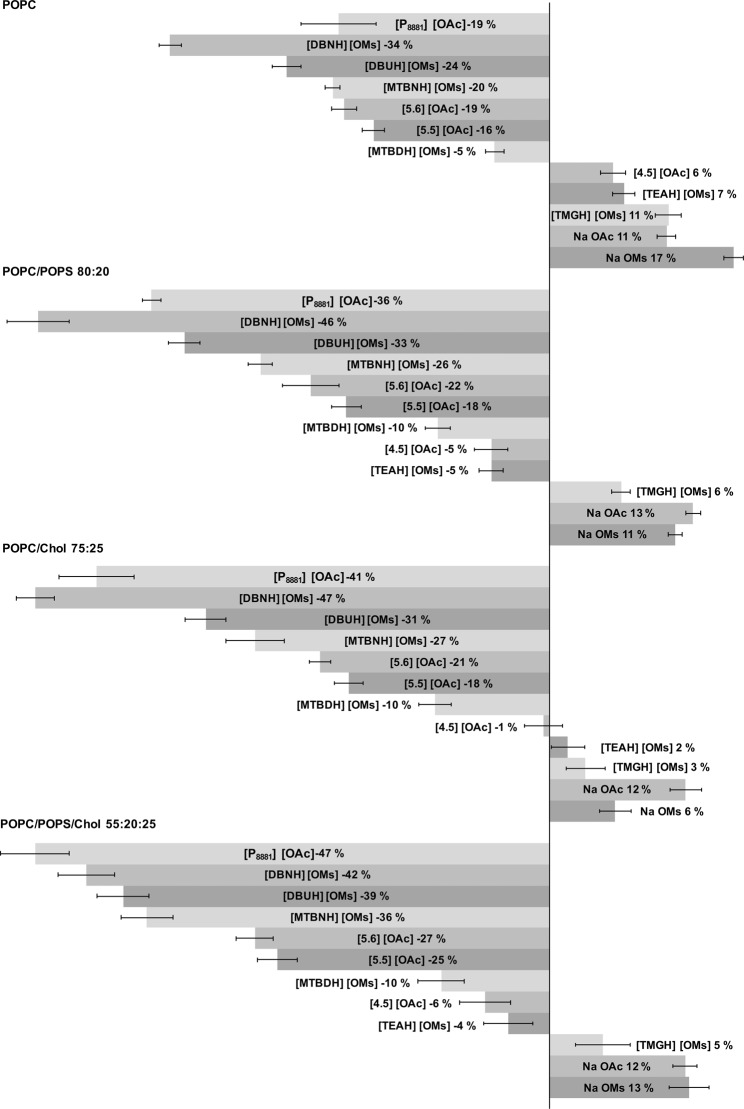
Relative change in anisotropy, *Δr*, measured using four different liposome compositions. The values were normalised relative to the anisotropy of pure liposomes, *i.e*., 0% means no interaction (horizontal axis). Positive values designate decreased rotational freedom within the lipid bilayer (acyl chains), whereas negative values designate increased rotational freedom, usually due to increased disorder of the chains. Either interaction may cause a toxic effect. Compound c = 50 mM, lipid c = 20 µmol.

Negative values for *Δr* are obtained when the *r*_*liposome*+*toxicant*_ decreases, relative to the anisotropy of *r*_*pure liposome*_. Negative *Δr* suggests that the rotational freedom within the bilayer is increasing and such an increase is due to the increased disorder of the bilayer, induced by the compounds permeating into the bilayer. In contrast, positive values are obtained when the *r*_*liposome*+*toxicant*_ increases. Positive *Δr* suggests that the rotational freedom within the bilayer is decreasing. Such decrease is caused by the small molecules permeating into the bilayer, but not considerably decreasing the organisation of the bilayer structure. Moreover such changes then cause subtle changes in the complex network of interactions within the bilayer, which further alternate the rotational freedom of the probe. The calculated *Δr* values are presented in Fig. 3.

[P_8881_][OAc], a highly toxic ionic liquid (EC_50_ = 2–3 µM), was selected as a positive control^25^. Since this IL, or rather the [P_8881_] cation, is surface active, the compound is known to be highly toxic towards *Vibrio Fischeri* and will efficiently penetrate into the bilayer structure, resulting in disruption of the membrane structure, as shown by our previous NPS study^25^. Accordingly, in contrast to the 50 mM concentration used for the other compounds, a considerably lower concentration of 0.2 mM was used to induce similar *Δr* values to the compounds of interest. The main purpose of the positive control was to highlight the effect of very toxic surface-active compounds on the liposomes and to show that the current method is valid also for such compounds. For [P_8881_][OAc] *Δr* values between −19% and −47% were obtained, depending on the liposome composition. This suggests that the permeation of the compound (mostly, the permeation of the lipophilic cation) into the bilayers can be detected using the current methodology.

NaOAc and NaOMs were used to investigate the effect of the anion on the bilayer structure and *Δr* values of 11–13% and 6–17% were obtained, respectively (Fig. 3). The result suggests that the anions are able to enter the bilayer structures, but do not disrupt the organisation. Based on their structures they most likely do not permeate deep into the hydrophobic acyl chain region, but more likely interact more with the head groups. However, large amount of such anions located in the head group region may induce reorganisation within the acyl chains and finally stimulate changes in rotational freedom of the probe. Due to the non-destructive interactions, these two compounds are also the least toxic ones. Interestingly, the presence or absence of POPS and the resulting net negative charge of the liposomes does not have a clear effect on the permeation of the anions into the bilayers. This may be due to the presence of a high number of both positive and negative charges in the solution, and therefore the surface charge of the liposomes does not play a significant role.

1,1,3,3-tetramethylguanidinium mesylate ([TMGH][OMs]) is the first compound that deviates considerably from the expected behaviour, when comparing the interaction data with the toxicity results (Figs 1 and 3). Based on the liposome interaction studies the *Δr* is 3–11%, depending on the liposome composition. This suggests that such a compound would be among the least toxic compounds, but based on the toxicity studies this compound is the second most toxic. However, the third least toxic compound [TEAH][OMs] instead follows the expected pattern with mean *Δr* values from −5% to +7%,. Among the spirocyclic acetates, 5-azoniaspiro[4.5]decane acetate ([4.5][OAc]) is the least toxic compound and also shows very minimal effect on the liposomes, with *Δr* values from −6% to +6%.

Overall, the remaining seven compounds, i.e. 1,8-Diazabicyclo[5.4.0]undec-7-enium mesylate ([DBUH][OMs]), [TMGH][OMs], 7-Methyl-1,5,7-triazabicyclo[4.4.0]dec-5-enium mesylate ([MTBDH][OMs]), a mixture of 5-Methyl-1,5,7-triazabicyclo-[4.3.0]non-6-enium and 7-Methyl-1,5,7-triazabicyclo[4.3.0]non-5-enium mesylate ([MTBNH][OMs]), 1,5-Diazabicyclo[4.3.0]non-5-enium mesylate [DBNH][OMs], 6-azoniaspiro[5.5]undecane acetate ([5.5][OAc]) and 6-azoniaspiro[5.6]dodecane ([5.6][OAc]), have very similar toxicities, with EC_50_ values around 5–15 mM (Fig. 1). However, the 5 min EC_50_ values, particularly for the spirocyclic acetates [5.6][OAc] and [5.5][OAc], are slightly higher than those for the remaining five most toxic compounds (Fig. 1). The subtle difference in toxicity between the aforementioned compounds, in contrast, shows as a somewhat more evident difference in *Δr*, particularly regarding the two- and three-component liposomes (Fig. 3). The remaining five most toxic compounds ([DBUH][OMs], [TMGH][OMs], [MTBDH][OMs], [MTBNH][OMs] and [DBNH][OMs]) have practically the same EC_50_ values, considering the error bars (Fig. 1). Additionally, for [DBUH][OMs], [TMGH][OMs], [MTBNH][OMs] and [DBNH][OMs] the *Δr* values coincide quite well with the toxicity data (Figs 1 and 3). The order of the compounds regarding the most toxic versus the most “interactive” is not the same. However, the differences between the EC_50_ values and the differences between *Δr* values are rather small. [MTBDH][OMs], however, clearly deviates from this trend. This IL, as mentioned, is among the most toxic compounds, but shows very low impact on the liposomes with *Δr* ranging from −5% to −10%.

The presence of POPS did not seem to have a conclusive effect on the interactions between the compounds and the liposomes. Comparing the results obtained with pure POPC liposomes and POPC/POPS liposomes, there is an increase in *Δr* (Fig. 3). The increased values are possibly due to electrostatic interaction between the cations and the anionic PS group. However, comparing the results between POPC/POPS and POPC/Chol, the *Δr* values are roughly the same. Again, there is a small increase in the *Δr* values for POPC/POPS/Chol relative to POPC/Chol. As mentioned (*vide supra*), the presence of large concentrations of anions and cations in the solution may diminish the effect of the liposome PS groups.

The reasons behind the fact that [MTBDH][OMs] and [TMGH][OMs] do not follow the trend shown by the other compounds, are not explainable in the context and scope of this study. However, the reasons may be speculated. The toxicity of [MTBDH][OMs] follows a common trend, since other heterobicyclic mesylates share very similar toxicities (Fig. 1). However, the impact on the bilayer organisation did not follow the same trend as the other accompanying heterobicyclic compounds (Fig. 3). The same behaviour was recorded for all four liposome compositions, so therefore the behaviour is most likely not an experimental artefact, but instead a true property of the compound. However, explanation of this behaviour would require more extensive studies, and as stated, this is not in the scope of this study. [TMGH][OMs], in contrast, showed a higher toxicity than expected, *e.g*. relative to the structurally similar [TEAH][OMs], even though the interactions with the membranes were very similar among these two compounds. This may be explained by a sensitivity of specific organisms to certain compounds, and this again suggests that here the mechanism of the toxicity is related to something else than permeation into or through the membranes of the bacteria.

Regarding the impact of the liposome composition (see Table 2 for compositions and Figs. 1 and 3 for results) on *Δr*, POPC/POPS/Chol 55:20:25 mol% liposomes seem to correlate the best with the toxicity results, if [TMGH][OMs] and [MTBDH][OMs] data is excluded. Here *Δr* is the largest for the very toxic positive control [P_8881_][OAc]. [DBNH], [DBUH], and [MTBNH] mesylates are among the most toxic compounds but also show the largest *Δr* values. [5.6][OAc] and [5.5][OAc] show somewhat lower toxicities and are interacting less with the liposomes. Further, the toxicity and *Δr* for [4.5][OAc] are clearly lower than for the aforementioned compounds. With [TEAH][OMs] the same trend continues. Finally, NaOAc and NaOMs both show very low toxicities and positive *Δr*.

Generally, in fluorescence anisotropy studies, the effect induced by the compounds depends greatly on the compound itself, the concentration of the compounds and on the liposome composition (not to mention the incubation temperature and the time) as illustrated by the present study and the examples below. In the present study the concentration of the compounds was in millimolar range (50 mM) in order to match better with the EC_50_ values measured for the compounds. Both the anion and cation seemed to permeate into the bilayer and most probably the effects of the anion and cation within the bilayer are additive. Up to 50% changes in anisotropy were recorded. In contrast, somewhat smaller changes of <10% in fluorescence polarisation (comparable to anisotropy) induced by 333 mM ethanol was recorded using phospholipids extracted from brain synaptosomal membranes^27^. Very similar changes were detected when the interactions of local anaesthetics at 0.2 mM concentration were investigated by using biomimetic liposomes composed of typical human cardiomyocyte mitochondrial membrane lipids *i.e*. cardiolipin, neutral and charged phospholipids, and cholesterol^28^. Similar concentrations were used for certain illicit drugs, and both positive and negative changes up to ~40% in polarisation were detected for some of the compounds when liposomes made of pure POPC and a mixture of POPC and 1-palmitoyl-2-oleoyl-*sn*-glycero-3-phospoglycerol (*i.e*. POPG for clarity) were used^29^. Therefore, comparison of results between studies is not very straightforward, if not exactly the same experimental conditions have been used.

## Conclusions

In the present study, the toxicity and the interactions of nine organic salts with biomimetic membranes, determined by fluorescence anisotropy, were investigated. Due to the non-surface-active nature and low toxicities of the compounds, techniques such as DSC or NPS are not sensitive enough to detect the permeation of such compounds into biomimetic lipid membranes. However, in the present study we show that fluorescence anisotropy is a suitable technique for determining such interactions. Finding a correlation between the toxicity towards a living organism, such as the *V. Fischeri* used in the present study and the non-living biomimetic membranes, was expected to be challenging. The fluorescence anisotropy technique is known to be susceptible to many variable factors, such as potential quenching of fluorescence, changes in fluorescence decay times and rotational factors of the probe induced by the changing environment of the probe, particularly when large quantities of non-lipidic compounds are introduced into the lipid system^18^. However, at this point we wanted to keep the present study as straightforward as possible and therefore, such factors are not yet considered. The interactions measured using fluorescence anisotropy correlated well with the toxicity values. Of course, one-to-one correlation was not expected, but fluorescence anisotropy spectroscopy seems to be a highly sensitive technique for following changes in the bilayer structures, induced by low-toxicity compounds.

**Figure 4:**
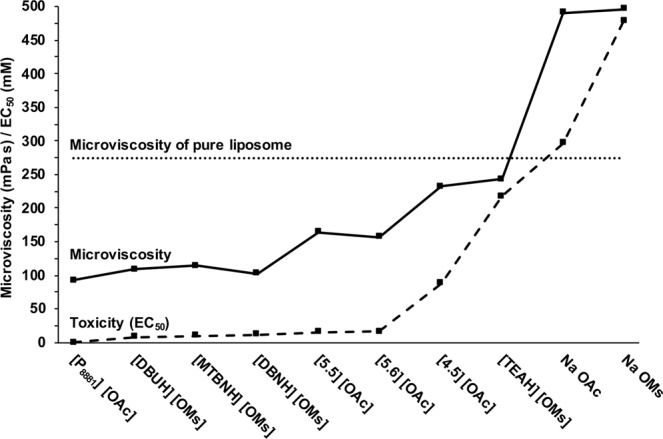
Comparison between microviscosity of POPC/POPS/Chol liposomes and toxicity (EC_50_) shows the common trend. Note, that here [TMGH][OMs] and [MTBDH][OMs] have been excluded, since they do not follow the trend. Microviscosity and EC_50_ share a common y-axis.

Excluding the two compounds ([MTBDH][OMs] and [TMGH][OMs]) that were not following the expected behaviour, the compounds which induced the most extensive interference in the bilayer organisation, also mostly showed the highest toxicity – and *vice versa*. Regarding the differing liposome compositions, cholesterol induced the biggest impact on the anisotropy of the pure liposomes. This was expected, since cholesterol is generally known to restrict the motion of the DPH probe, due to the space-filling between the acyl chains. Otherwise, the liposome composition did not seem to have a large practical effect on the interactions. With cholesterol-containing liposomes, the microviscosity remains above the 100 mPa·s threshold, typical for biological lipid membranes, even when exposed to the most destructive compound [P_8881_][OAc]. Whether the presence of cholesterol has any practical effect on the model membranes suitability to function as a toxicity model is currently inconclusive. However, POPC/POPS/Chol liposomes seem to correlate the best with the toxicity results. Figure 4 summarises the main result of the study by visualising the common trend in anisotropy-derived microviscosity for the POPC/POPS/Chol liposomes and the toxicity (*i.e*. 5 min EC_50_ values), when [MTBDH][OMs] and [TMGH][OMs] have been excluded. The trend shown by the graph suggests that the decreased order, *i.e*. decreased microviscosity, correlates with the increased toxicity, *i.e*. decreased EC_50_.

Our goal is to achieve a deeper understanding of the relationship between the structures of compounds and their toxicities. However, detecting the interactions between relatively small non-surface-active ILs or IL-like compounds and *in vitro* model membranes has been a challenge. The importance of the present study lies in the finding that permeation of such compounds into the membrane could be detected. For many compounds the interaction with model membranes and the toxicity correlated relatively well. New and more sensitive, yet at the same time robust methods are needed to investigate how the structure of low-toxicity compounds affects their toxicities. The present study shows the potential of using fluorescence anisotropy for such studies.

## Materials and Methods

### Chemicals

1-Palmitoyl-2-oleyl-*sn*-glycero-3-phosphocoline (POPC), 1-palmitoyl-2-oleyl-*sn*-glycero-3-phosphoserine sodium salt (POPS) and bovine wool cholesterol (Chol) were purchased from Avanti Polar Lipids (Alabaster, AL, USA). 1,6-Diphenyl-1,3,5-hexatriene (DPH) and sodium mesylate were acquired from Sigma-Aldrich (Steinheim, Germany). Chloroform was from VWR International (Leuven, Belgium). Water-free sodium acetate was from Riedel-de Haën (Seelze, Germany).

### Synthesis of ionic liquids and spirocyclic compounds

Table 1 shows the systematic names and structures of the nine synthesised compounds. Detailed synthetic procedures can be found in this article’s supplementary information. Additionally, three reference compounds were included: sodium acetate and sodium mesylate were studied in order to reveal the impact of the anion, whereas ([P_8881_][OAc]) was selected as a high-toxicity reference ionic liquid, found to be effective in biomass processing^30^.

### Toxicity determinations

The toxicities of the compounds were determined by measuring the median effective concentration (EC_50_) for each compound. This was done using *Vibrio Fischeri* bacteria and a Microtox Model 500 analyser (Modern Water plc, Guildford, UK) according to the manufacturer’s protocol. The compounds under investigation and the bacteria were incubated for 5 min and 15 min in order to determine an EC_50_ value for both time points. Based on the data, the EC_50_ values were determined by the software-based fitting using the MicrotoxOmni 4.2 software. Typically, four differing concentration points were enough to determine the EC_50_ value. However, for certain compounds, namely [TMGH][OMs], [DBNH][OMs] and [TEAH][OMs], the fitting performed by the MicrotoxOmni software did not pass our quality standards (R^2^ > 0.95). Therefore, a higher number of concentration points were measured, and fitting was completed using ‘Origin’ (OriginPro 2018b, OriginLab Corporation, Northampton, MA, USA). The fitting was performed using the *Dose-Response curve with variable Hill slope* -function of the software. The optimisation algorithm was *Levenberg Marquardt*. An example fit for data measured for [TEAH][OMs] is shown in Fig. S2 in Supplementary Information.

### Preparation of liposomes

The lipids and the DPH probe were dissolved in chloroform as stock solutions and the stocks were mixed to give the target molar proportions for each liposome composition (Table 2). After mixing the chloroform lipid stocks, the lipid mixture was dried as a thin film on test tube walls by evaporating the chloroform using a flow of air, until no solvent was visible. The dried thin film of lipids was kept under vacuum for at least 12 h in a desiccator, in order to remove any traces of solvent. Ultra-pure water was added to reach the target lipid concentration of 4.0 mM. The ratio of DPH and lipids was 1:499. The aqueous mixture of DPH, lipids and water were incubated under agitation for 60 min at 65 °C and 75 °C to form the non-cholesterol-containing and cholesterol-containing multilamellar liposomes, respectively. Subsequently, the 4.0 mM liposome stock dispersions were extruded through 100 nm pore-size polycarbonate filters 19 times using an Avanti mini-extruder (Avanti Polar Lipids, Alabaster, AL, USA) in order to obtain unilamellar liposomes.

### Steady-state fluorescence anisotropy measurements

The fluorescence anisotropy values were measured using a Horiba Jobin Yvon (Northampton, UK) FluoroMax-4 spectrofluorometer, in L-format. The emission and the excitation side-polarizers were always set to the same value (8–10 nm) so that the photon counts/second at the detector were as recommended by the manufacturer (from 500 000 to 2 000 000 counts/second). The excitation and emission wavelengths were 360 nm and 450 nm, respectively. The measurements were performed at 21 ± 1 °C. Physiological temperature was not used, since the interactions between the living organism (*Vibrio Fischeri*) also takes place at a non-physiological temperature of 15 °C. The maximum 7 °C difference (15 °C vs. 22 °C) was considered acceptable. A reference value for the pure liposomes was measured daily. In order to study the interaction between the compounds and the liposomes, the pure liposomes were mixed with the compounds so that the final concentration of the lipids was 20 µM and that of the studied compounds was 50 mM. For the highly toxic positive control, *i.e*. [P_8881_][OAc], the lower concentration of 0.2 mM was selected. Both concentrations were selected by trial and error. After mixing the liposomes and the compound, the mixture was briefly vortexed and then incubated at RT for 5 min, preceding the measurement.

## Supplementary information

Supplementary information

## Data Availability

The datasets generated and analysed during the current study are available from the corresponding authors on reasonable request.
