# Supplementary material for: Interactions of Ionic Liquids and Spirocyclic Compounds with Liposome Model Membranes. A Steady-State Fluorescence Anisotropy Study
Source: Sci Rep. 2019 Dec 4;9:18349. doi: 10.1038/s41598-019-53893-w (PMC6892914; doi:10.1038/s41598-019-53893-w)
Supplement: Supplementary file 1 — Supplementary information [file 41598_2019_53893_MOESM1_ESM.pdf]

INTERACTIONS OF IONIC LIQUIDS AND SPIROCYCLIC COMPOUNDS WITH LIPOSOME MODEL MEMBRANES. A STEADY-STATE FLUORESCENCE ANISOTROPY STUDY

Supplementary information

Antti H. Rantamäki*, Wen Chen, Paulus Hyväri, Jussi Helminen, Gabriel Partl, Alistair W.T. King, Susanne K. Wiedmer*

Department of Chemistry, A.I. Virtasen aukio 1, P.O. Box 55, FI-00014 University of Helsinki, Finland

*Correspondence: Dr. Antti H. Rantamäki & Dr. Susanne K. Wiedmer
antti.rantamaki@helsinki.fi
susanne.wiedmer@helsinki.fi

SYNTHESIS OF IONIC LIQUIDS AND SPIROCYCLIC COMPOUNDS

5-Azoniaspiro[4.5]decane bromide: 4.26 g (50.0 mmol, 99% purity) of piperidine, 10.8 g (50.0 mmol 99% purity) of 1,4-dibromobutane and 8.4 g (100.0 mmol) of $NaHCO_3$ were dissolved/dispersed in 100 mL of EtOH and the resulting mixture refluxed for 36 h. After that, EtOH was evaporated and the product extracted twice in 100 mL of boiling MeCN each, filtered while hot and the solvent evaporated. Subsequently, the residue was taken up in 15 mL of MeOH, followed by reprecipitation via addition of 100 mL of Et_2O under vigorous stirring. The product was filtered off, washed with Et_2O twice (50 mL each) and dried under reduced pressure. 10.0 g (91% yield) of white, powdery 5-azoniaspiro[4.5]decane bromide were isolated.

^{1}H NMR (600 MHz, D_2O) δ 3.57 (t, J = 6.9 Hz, 4H), 3.40 (t, J = 6.0 Hz, 4H), 2.21 (m, 4H), 1.92 (m, 4H), 1.72 (p, J = 6.0 Hz, 2H) ppm.

5-Azoniaspiro[4.5]decane acetate, [4.5][OAc]: 4.0 g (18.17 mmol) of 5-azoniaspiro[4.5]decane bromide and 3.03 g (18.17 mmol) of silver acetate were dissolved/dispersed in 50 mL of MeOH and stirred for 16 h at RT and light-protection. Subsequently, generated AgBr was filtered off over Celite 545, followed by evaporation of the solvent and drying the product *in vacuo*. 3.52 g (97% yield) of almost colorless 5-azoniaspiro[4.5]decane acetate were isolated. Prior to the toxicity tests, [4.5][OAc] was dissolved in water and syringe-filtered (0.45 µm) in order to remove any residual particulate silver (Ag content was confirmed to be below the l.o.d. (10 ppb) using MP-AES).

^{1}H NMR (600 MHz, DMSO-d_6) δ 3.51 (t, J = 7.3 Hz, 4H), 3.36 (t, J = 6.0 Hz, 4H), 2.03 (m, 4H), 1.76 (m, 4H), 1.55 (p, J = 6.0 Hz, 2H), 1.49 (s, 3H) ppm. ^{13}C NMR (151 MHz, DMSO-d_6) δ 171.96, 61.49 (2C), 59.06 (2C), 26.43 (2C), 20.86 (2C), 20.82, 20.76 ppm. m.p. (DSC) = 100-135 °C

6-Azoniaspiro[5.5]undecane bromide: To 600 mL of chloroform, 86.0 g (100 mL, 1.0 mol, 99% purity) of piperidine, 234.6 g (139 mL, 1.0 mol, 98% purity) of 1,5-dibromopentane and 130.6 g (175 mL, 1.0 mol, 99% purity) of diisopropylethylamine were added and the mixture heated under reflux for 15 h, during which time the product precipitated from solution. Subsequently, the mixture was filtered while still warm and the product washed twice with 200 mL of chloroform each and once with 200 mL of acetone. Finally, the product was dried *in vacuo* to give 232 g (991 mmol, 99% yield) of free-flowing, pure-white, powdery 6-azoniaspiro[5.5]undecane bromide.

^{1}H NMR (300 MHz, D_2O) δ 3.42 (t, J = 6.0 Hz, 8H), 1.89 (p, J = 6.0 Hz, 8H), 1.71 (p, J = 6.0 Hz, 4H) ppm.

6-Azoniaspiro[5.5]undecane acetate, [5.5][OAc]: A mixture of 3.29 g (14.05 mmol) of 6-azoniaspiro[5.5]undecane bromide, 2.34 g (14.05 mmol) of silver acetate and 50 mL of MeOH was stirred for 16 h, covered in aluminium foil. Subsequently, precipitated AgBr was filtered off over Celite 545, the filtrate evaporated and the residue dried *in vacuo*. 2.93 g (98% yield) of white to off-white 6-azoniaspiro[5.5]undecane acetate were isolated. Prior to the toxicity tests, [5.5][OAc] was dissolved in water and syringe-filtered (0.45 µm) in order to remove any residual particulate silver (Ag content was confirmed to be below the l.o.d. (10 ppb) using MP-AES).

^{1}H NMR (600 MHz, DMSO-d_6) δ 3.42 (t, J = 6.0 Hz, 8H), 1.74 (p, J = 6.0 Hz, 8H), 1.54 (p, J = 6.0 Hz, 4H), 1.49 (s, 3H) ppm. ^{13}C NMR (151 MHz, DMSO-d_6) δ 171.97, 58.09 (4C), 26.43 (4C), 21.17 (2C), 18.69 ppm. m.p. (melting point apparatus) = 84-130 °C

6-Azoniaspiro[5.6]dodecane bromide: 5.0 g (50.0 mmol, 99% purity) of azepane, 11.85 g (50.0 mmol, 97% purity) of 1,5-dibromopentane and 8.4 g (100.0 mmol) of $NaHCO_3$ were dissolved/dispersed in 100 mL of EtOH and refluxed for 24 h. Afterwards, the solvent was evaporated and the product extracted in 100 mL of boiling MeCN four times, filtered while hot and the solvent evaporated. Subsequently, the residue was dissolved in 15 mL of MeOH, followed by reprecipitation via addition of 100 mL of Et_2O under vigorous stirring. The product was filtered off, washed with Et_2O twice (50 mL each) and dried *in vacuo*. 10.0 g (81% yield) of white, powdery 6-azoniaspiro[5.6]dodecane bromide were isolated.

^{1}H NMR (600 MHz, D_2O) δ 3.51 (m, 4H), 3.40 (t, J = 5.7 Hz, 4H), 1.92 (m, 8H), 1.73 (m, 6H) ppm.

6-Azoniaspiro[5.6]dodecane acetate, [5.6][OAc]: 4.0 g (16.1 mmol) of 6-azoniaspiro[5.6]dodecane bromide and 2.69 g (16.1 mmol) of silver acetate were dissolved/dispersed in 50 mL of MeOH and stirred for 16 h at RT and under light-protection. Afterwards, generated AgBr was filtered off over Celite 545, the methanolic solution evaporated and the product dried *in vacuo*. 3.55 g (97% yield) of off-white 6-azoniaspiro[5.6]dodecane acetate were isolated. Prior to the toxicity tests, [5.6][OAc] was dissolved in water and syringe-filtered (0.45 µm) in order to remove any residual particulate silver (Ag content was confirmed to be below the l.o.d. (10 ppb) using MP-AES).

^{1}H NMR (600 MHz, DMSO-d_6) δ 9.41 (s, 1H), 3.45 (m, 4H), 3.36 (t, J = 6.0 Hz, 4H), 1.80 (m, 4H), 1.75 (t, J = 6.0 Hz, 4H), 1.58 (m, 4H), 1.54 (m, 2H), 1.49 (s, 3H) ppm. ^{13}C NMR (151 MHz, DMSO-d_6) δ 171.90, 61.28 (2C), 60.21 (2C), 27.03 (2C), 26.42 (2C), 20.70, 20.27 (2C), 19.38 ppm. No melting point, viscous liquid.

[DBNH][OMs]: To 50.25 g (404.7 mmol, 50.0 mL) of Ar-flushed DBN, stirred at 4 °C, 38.89 g (404.7 mmol, 26.28 mL) of HOMs were slowly added. Upon solidification, the mixture was brought to RT, then gently heated using a heat gun until acid addition was complete. Subsequently, the mixture was heated to 130 °C for 30 min in order to ensure complete mixing of the reactants. Quantitative amounts of colourless, solid [DBNH][OMs] were isolated.

^{1}H NMR (600 MHz, DMSO-d_6) δ 8.06 (s, 1H), 3.60 (t, J = 7.6 Hz, 2H), 3.38 (t, J = 5.8 Hz, 2H), 3.28 (t, J = 5.8 Hz, 2H), 2.79 (t, J = 7.6 Hz, 2H), 2.34 (s, 3H), 2.02 (p, J = 7.6 Hz, 2H), 1.90 (p, J = 5.8 Hz, 2H) ppm. ^{13}C NMR (151 MHz, DMSO-d_6) δ 163.78, 52.94, 41.92, 39.69, 37.73, 29.78, 18.31, 18.26 ppm. m.p. (DSC) = 98 °C.

[DBUH][OMs]: To 7.61 g (50.0 mmol) of Ar-flushed DBU, 4.81 g (50.0 mmol) of HOMs were added and the resulting exothermic mixture stirred for 30 min. This gave quantitative amounts of liquid, highly viscous [DBUH][OMs], which crystallised over time when stored.

^{1}H NMR (600 MHz, DMSO-d_6) δ 3.55 (m, 2H), 3.48 (t, J = 5.8 Hz, 2H), 3.25 (t, J = 5.8 Hz, 2H), 2.66 (m, 2H), 2.32 (s, 3H), 1.92 (p, J = 5.8 Hz, 2H), 1.71 – 1.57 (m, 6H) ppm. ^{13}C NMR (151 MHz, DMSO-d_6) δ 165.35, 53.33, 47.84, 39.71, 37.61, 31.61, 28.18, 25.88, 23.31, 18.86 ppm. m.p. (melting point apparatus) = 37-41 °C

Mixture of [MTBNH][OMs] isomers: To 7.5 g (53.9 mmol) of an Ar-flushed mixture of 5-methyl-1,5,7-triazabicyclo[4.3.0]non-6-ene and 7-methyl-1,5,7-triazabicyclo[4.3.0]-non-5-ene (MTBN), 5.18 g (53.9 mmol) of HOMs were slowly added under stirring. Subsequently, the solidified mixture was heated to 120 °C for 15 min in order to ensure complete mixing of the reactants. Quantitative amounts of slightly orange, solid [MTBNH][OMs] were isolated.

^{1}H NMR (600 MHz, DMSO-d_6) δ 8.68 (s, 1H), 8.38 (s, 1H), 3.66 – 3.48 (m, 8H), 3.32 – 3.19 (m, 8H), 2.97 (s, 3H), 2.87 (s, 3H), 2.32 (s, 6H), 1.98 (p, J = 5.9 Hz, 2H), 1.90 (p, J = 5.9 Hz, 2H) ppm. m.p. (DSC) = 105-106 °C.

[MTBDH][OMs]: To 5.00 g (32.6 mmol) of Ar-flushed N-methyl-1,5,7-triazabicyclo[4.4.0]dec-5-ene (MTBD), 3.13 g (32.6 mmol) of HOMs were slowly added under stirring. Subsequently, the solidified mixture was heated to 130 °C for 15 min in order to ensure complete mixing of the reactants. Quantitative amounts of colourless, solid [MTBDH][OMs] were isolated.

^{1}H NMR (600 MHz, DMSO-d_6) δ 7.71 (s, 1H), 3.30 (q, J = 6.0 Hz, 4H), 3.27 (t, J = 6.0 Hz, 2H), 3.24 (td, J = 6.0, 3.2 Hz, 2H), 2.92 (s, 3H), 2.32 (s, 3H), 1.93 (p, J = 6.0 Hz, 2H), 1.86 (p, J = 6.0 Hz, 2H) ppm. ^{13}C NMR (151 MHz, DMSO-d_6) δ 150.70, 47.50, 46.99, 46.50, 39.69, 38.34, 36.98, 20.37, 20.21 ppm. m.p. (DSC) = 123 °C.

[TEAH][OMs]: To 151.8 g (1.5 mol) of NEt_3 (TEA), 144.2 g (1.5 mol) of HOMs were slowly added under stirring and Ar atmosphere. After the addition was completed, another 5 mL of TEA were added and the viscous mixture stirred for 30 min, followed by drying of the product *in vacuo*. Over time, the slightly orange product solidified to give quantitative amounts of [TEAH][OMs].

^{1}H NMR (600 MHz, DMSO-d_6) δ 9.20 (s, 1H), 3.09 (dq, J = 7.3, 4.6 Hz, 6H), 2.39 (s, 3H), 1.18 (t, J = 7.3 Hz, 9H) ppm. ^{13}C NMR (151 MHz, DMSO-d_6) δ 45.73 (3C), 39.70, 8.51(3C) ppm. m.p. (DSC) = 28 °C.

[TMGH][OMs]: To 11.52 g (100.0 mmol) of Ar-flushed TMG, 9.61 g (100.0 mmol) of HOMs were slowly added under stirring. Subsequently, the solidified mixture was heated to 120 °C for 20 min in order to ensure complete mixing of the reactants. Quantitative amounts of colourless, solid [TMGH][OMs] were isolated.

^{1}H NMR (600 MHz, DMSO-d_6) δ 7.81 (s, 2H), 2.89 (s, 12H), 2.32 (s, 3H) ppm. ^{13}C NMR (151 MHz, DMSO-d_6) δ 160.95, 39.74, 39.35 (4C) ppm. m.p. (melting point apparatus) = 117-125 °C

Methyltrioctylphosphonium acetate, [P_{8881}][OAc]: Labafzadeh *et al.*[30]

TOXICITY DETERMINATIONS

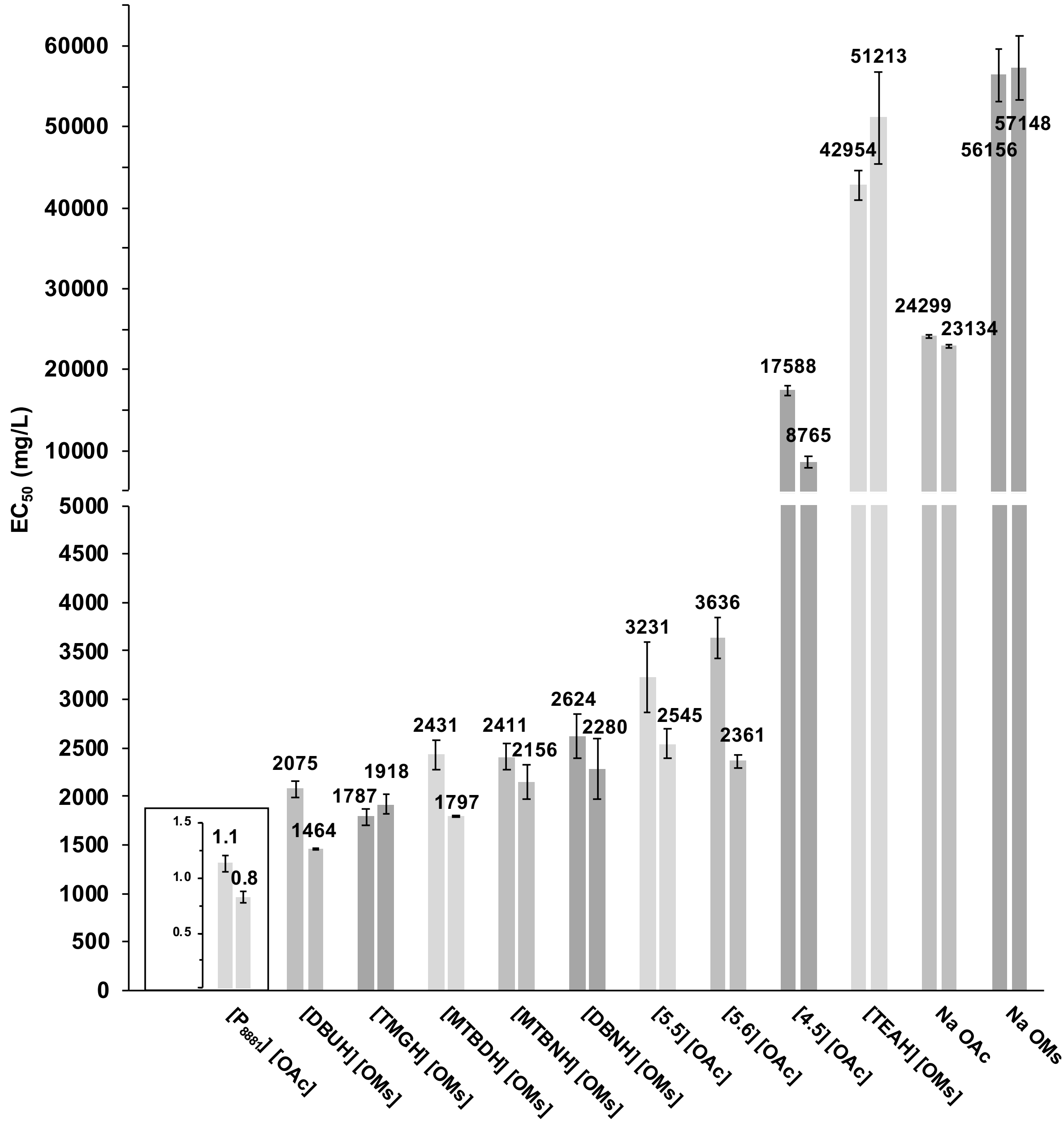

Figure S1. The median effective concentrations EC_{50} for the ionic liquids and the spirocyclic compounds measured using *Vibrio Fischeri* bacteria. The EC_{50} values are the mean of two duplicate measurements ± standard deviation. Note the different scale for $[P_{8881}]$[OAc] and the change of the scale along the vertical axis.

Left-hand bar, 5 min exposure; Right-hand bar, 15 min exposure

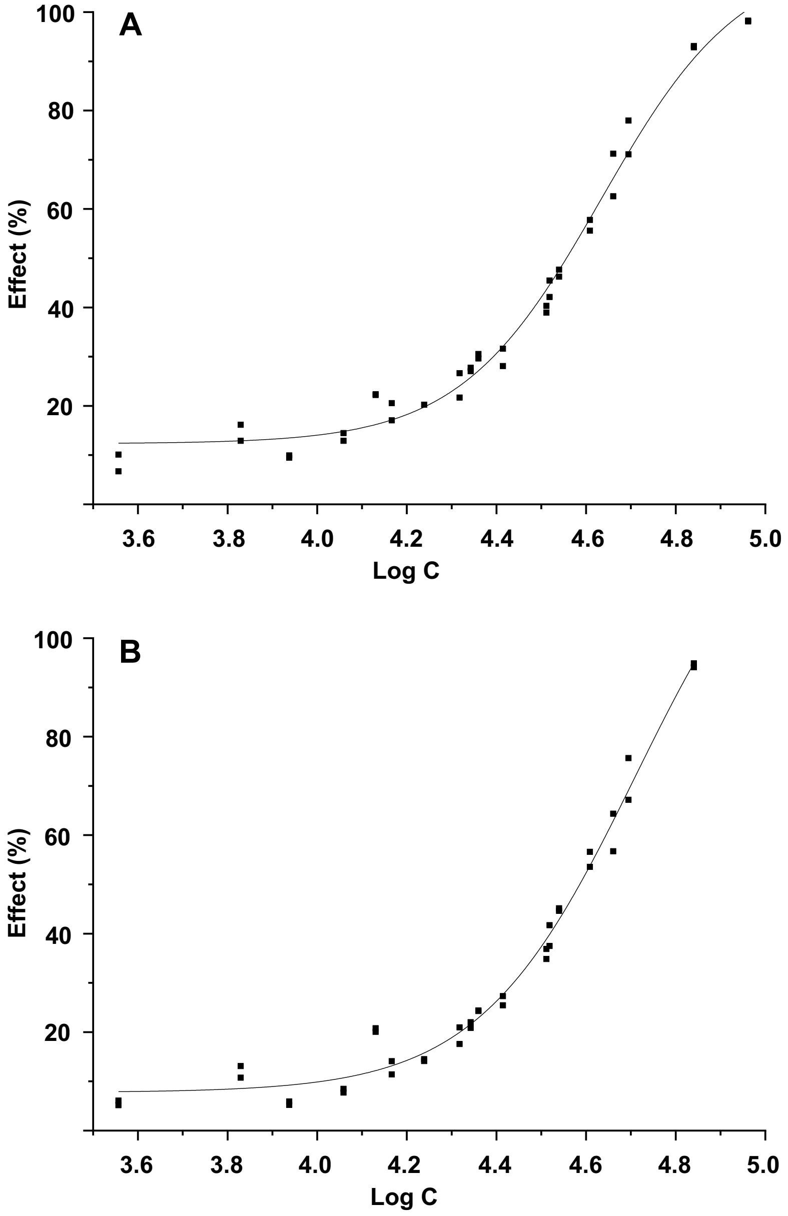

Figure S2. Example fit of [TEAH][OMs] toxicity data by the *Dose-Response curve with variable Hill slope*-function (OriginPro 2018b, OriginLab Corporation, Northampton, MA, USA). A) 5 min data, B) 15 min data.
